# Portable Devices to Induce Lucid Dreams—Are They Reliable?

**DOI:** 10.3389/fnins.2019.00428

**Published:** 2019-05-08

**Authors:** Sérgio A. Mota-Rolim, Achilleas Pavlou, George C. Nascimento, John Fontenele-Araujo, Sidarta Ribeiro

**Affiliations:** ^1^Brain Institute, Federal University of Rio Grande do Norte, Natal, Brazil; ^2^Physiology and Behavior Department, Federal University of Rio Grande do Norte, Natal, Brazil; ^3^Onofre Lopes University Hospital, Federal University of Rio Grande do Norte, Natal, Brazil; ^4^Psychology Department, University of Essex, Colchester, United Kingdom; ^5^Biomedical Engineer Department, Federal University of Rio Grande do Norte, Natal, Brazil

**Keywords:** lucid dreaming, rapid eye movement sleep, dreams, sleeping mask, headband

## Introduction

One of the main current challenges in lucid dreaming (LD) research is to develop a simple and reliable way to induce it (Stumbrys et al., 2012). This is because, for most people, LD is very pleasurable but also very rare (LaBerge and Rheingold, 1990; Mota-Rolim et al., 2013). Along with its recreational nature, LD also has potential clinical applications, such as the treatment of recurrent nightmares in post-traumatic stress disorder (Aurora et al., 2010; Mota-Rolim and Araujo, 2013; Morgenthaler et al., 2018). This has attracted the attention of high-tech companies, which have been launching portable LD induction devices commercially available to the general public.

**Figure 1:**
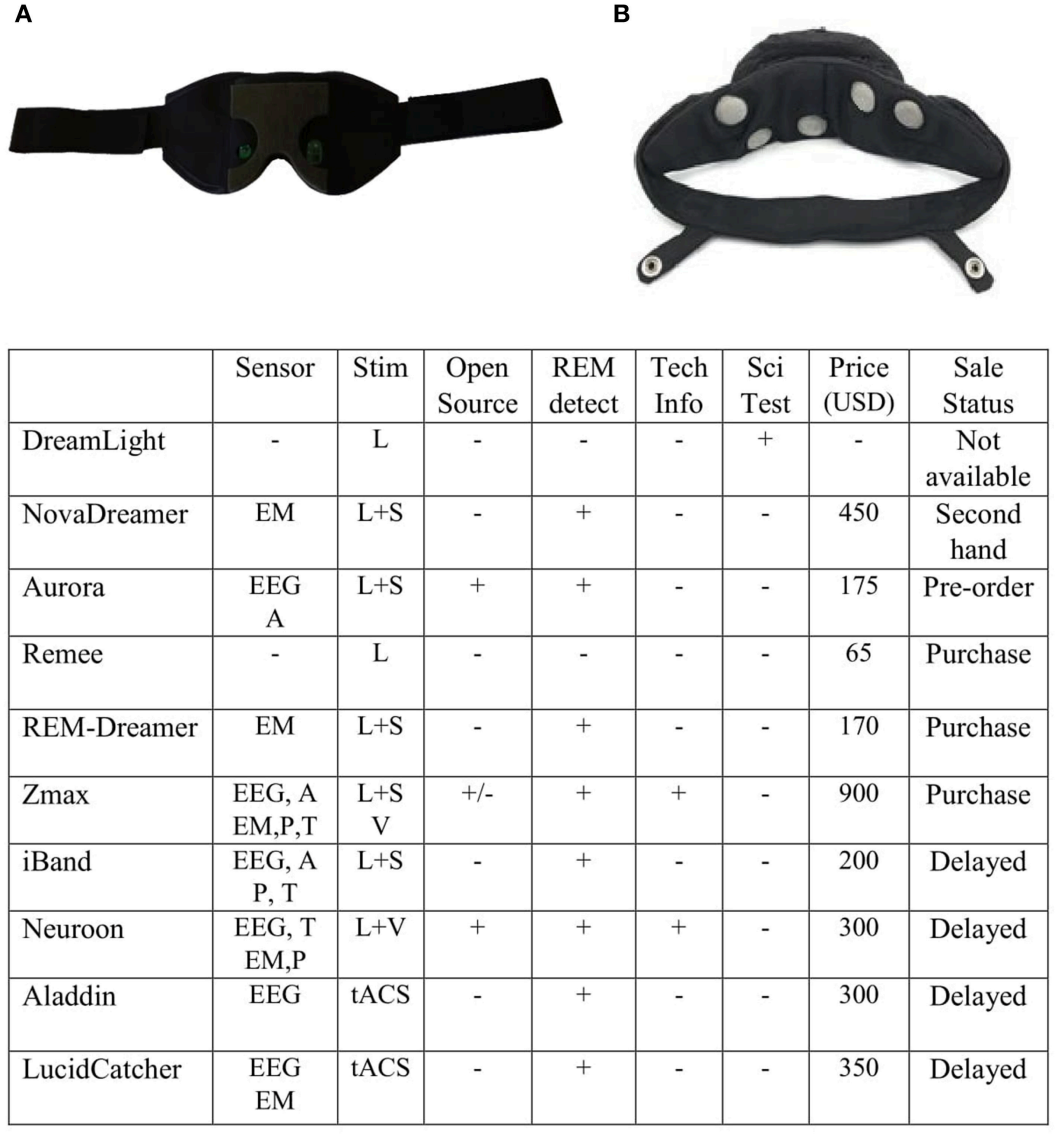
Up: Internal views of **(A)** REM-Dreamer sleeping mask and **(B)** LucidCatcher headband (images reproduced with permission from ELI Company and Luciding Inc). Down: Table comparing the devices. Stim, Stimulus; REM detect, online REM sleep detection; tech info, technological information; sci test, scientifically tested; L, light; S, sound; V, vibration; EM, eye movement; A, accelerometer; T, temperature; P, pulse oximeter; tACS, transcranial alternating current stimulation. Neuroon, iBand, Aurora, and Aladdin electrodes are placed on Fp1/Fp2 referenced to FpZ; ZMax electrodes are placed on AF7/AF8 referenced to FpZ; and LucidCatcher electrodes are placed on Fp1/Fp2 referenced to TP9/TP10.

This equipment captures electroencephalographic (EEG) activity for the online detection of rapid eye movement (REM) sleep, the sleep stage associated with typical dreaming (Aserinsky and Kleitman, 1953; Dement and Kleitman, 1957; for review, see Hobson et al., 2000). To induce lucidity, most devices provide visual, auditory, and/or tactile stimuli as sensory cues, which can become incubated into the dream content to alert dreamers that they are dreaming but without waking them up (LaBerge et al., 1981a; LaBerge and Levitan, 1995). Other devices provide transcranial alternating current stimulation (tACS) of the frontal cortex (Voss et al., 2014). Here we review 10 such devices: DreamLight, NovaDreamer, Aurora, Remee, REM-Dreamer, ZMax, Neuroon, iBand, LucidCatcher, and Aladdin (Figure 1).

## The pioneers: DreamLight and NovaDreamer

In the early 1980s, neuroscientists tried to induce LD by verbal suggestion (LaBerge et al., 1981a), musical tones (Kueny, 1985), tactile stimuli (Rich, 1985), and olfactory stimuli (LaBerge et al. unpublished data). In 1987, Stephen LaBerge conducted the first study on inducing LD by light stimulation during REM sleep: of 28 volunteers, 17 (61%) reported having experienced at least one LD episode (LaBerge, 1987).

With the success of light stimulation, LaBerge and Levitan (LaBerge and Levitan, 1995) tested for the first time a portable computerized biofeedback device, named DreamLight. Lights were used during REM sleep in 14 subjects for 4–24 nights. As a way to control for the placebo effect, lights were delivered on alternate nights, without the volunteers' knowledge. Eleven subjects (78%) reported 32 LD episodes: 22 happened on nights with the light cues and 10 on nights without them. Besides, the volunteers reported seeing the cues in their dreams significantly more often on light-cue nights compared to non-light-cue nights (73 vs. 9, respectively).

Following these experiments, LaBerge and co-workers from the Lucidity Institute released the first commercialized product to induce LD: the sleeping mask NovaDreamer. This device detects REM sleep automatically and delivers flashing lights to incubate these stimuli into the dream, as a cue to induce lucidity. The mask was available in the market until 2004, when its production was discontinued. In 2009, the Lucidity Institute reported working on a new NovaDreamer, which would be released in 2016, but since then, no update has been announced.

## The modern devices

### Products That Are Available in the Market

Aurora was the first headband launched on a crowdfunding platform. Its campaign started in December 2013, asking for US$ 90,000, and in 40 days, they raised almost US$ 240,000. Aurora has electrodes for EEG oscillation detection and accelerometers that track body movements. According to their site: “Our experiments with real-time sleep stage detection have proven very accurate with 90% of our experimental subjects”; however, the developers do not provide enough scientific information on how their algorithm calculates accuracy, nor make the data supporting this claim accessible. They also admit some limitations of the method and posted as a disclaimer that the “REM-detection algorithm is not yet perfect.” To date, the system is not available for immediate purchase but can be ordered. The Aurora platform is open-source and thus allows users to contribute in developing the system.

Remee is the cheapest sleeping mask and the only one that does not use online sleep stage detection. According to their site: “Using a series of smart timers, light patterns are displayed throughout the night…” This means that lights can appear during REM sleep or during the other sleep stages: sleep onset (N1), superficial sleep (N2), and deep sleep (N3). It is known that LD happens predominantly during REM sleep (LaBerge et al., 1981b, 1986) and less often during N1 and N2 sleep stages (LaBerge, 1980a,b, 1990; LaBerge et al., 1981a; Dane and Van de Caslte, 1984; Stumbrys and Erlacher, 2012; Mota-Rolim et al., 2015). However, if lights appear during N3, they will most probably fail to induce LD, since there are no reports of LD during this sleep stage. Besides, this mask may potentially impair sleep quality by disturbing the slow waves that occur during N3, which are related to the homeostatic restoration function of sleep (Benington and Heller, 1995).

The REM-Dreamer device (Figure 1A) has two features among all masks. First, it can induce lucidity by recording and playing voice messages, such as the user saying “I am dreaming,” for instance, which can incubate into dreams (LaBerge et al., 1981a). Second, it allows communication between the dreamer and the machine. This feature is based on the ideas that (1) subjective eye movements during dreaming correlate with objective eye movements (that is, real eyeball rotations), as postulated by the “scanning hypothesis” (Roffwarg et al., 1962; for review, see Arnulf, 2011; LaBerge et al., 2018a,b); and (2) it is possible to voluntarily move the eyes to indicate dream lucidity (Hearne, 1978; LaBerge, 1980a,b). Thus, when the dreamer perceives the cues, the dreamer can move the eyes in such a predetermined manner that the device would sense this movement and stop generating the stimuli. The sleeping mask utilizes infrared sensors to detect when the user is in REM sleep; however, not enough technical information is available on how the algorithm implements this.

Hypnodyne's ZMax became available for sale in 2018 and is the most expensive device nowadays. ZMax is a sleep-monitoring headband that delivers light, vibrotactile, and auditory stimuli, and also allows audio-recording of dream experiences. ZMax is currently being tested in various universities and scientific institutions around the world. The device monitors sleep through two frontal sensors, which capture brain activity and ocular movements. In contrast to other devices that use dry EEG sensors, ZMax uses proprietary disposable solid hydrogel electrodes. In addition, it includes sensors for heart rate (acquired through a photoplethysmogram; PPG), temperature, ambient light, sound, and body movements. ZMax features offline autoscoring and online REM sleep detection algorithms, whose technical information is available and comprehensive. The accuracy of ZMax relies in part on individual EEG phenotype detection. To do this, REM sleep classification is initially delayed for 2 h, a period that will usually include at least the first sleep cycle. When this time has elapsed, the system analyzes the sleep data collected thus far and extracts a brief phenotype description of the individual. The result is saved in a subject-specific file, which can be loaded for subsequent trials, before data collection. Importantly, ZMax's online algorithms, whether for REM sleep detection or for stimulus protocols, require a computer to be connected through a wireless connection dongle because the algorithmic computations occur on the computer and are transmitted back to ZMax. Despite ZMax not being open-source, it allows for the scripting of several functions in JavaScript for custom stimuli. ZMax can also be interfaced with various other programming languages (MATLAB, Python, PHP, C++, Java, etc.) through an exposed TCP/IP^1^ data socket.

### Products That Are Under Development

Neuroon includes a mobile app dream diary, which is a good method to increase dreams and LD recall (LaBerge and Rheingold, 1990). It is open-source and also was launched in a crowdfunding platform: they asked for US$100,000 in pledges in June 2017, and 1 month later, they achieved almost $360,000. Besides measuring EEG activity, Neuroon has a pulse oximeter (PPG) and sensors for temperature and ocular movements, which would allow for online detection of REM sleep. The technical documentation of Neuroon is accessible; however, despite claiming the use of established techniques to induce LD (i.e., visual and tactile stimulation; Paul et al., 2014), the product is yet to be scientifically tested. More recently, the company behind Neuroon has filed for bankruptcy, and its future is thus uncertain.

iBand is the device that got the most crowdfunding support. They started their campaign in September 2016, asking for €50,000, and in 44 days received around €64,500. This headband has sensors that measure brain rhythms, body movement, temperature, and heart rate, and claims to analyze them through an “auto-learning software algorithm.” However, its platform is not open-source, and the technical details of this algorithm are not available.

LucidCatcher (Figure 1B) and Aladdin are the only headbands that promise to induce LD using tACS of the frontal region. Since frontal gamma power (~40 Hz) increases during LD (Mota-Rolim et al., 2008, 2010; Voss et al., 2009), Voss et al. (2014) used a low current to induce gamma activity on the frontal region during REM sleep and successfully increased self-awareness subjective scores during dreaming. Despite the claim that the Voss et al. (2014) study “was replicated by Aladdin in an IRB-approved clinical study,” we could not find these data nor any related scientific publication. Importantly, there has not been a published reproduction of Voss et al. (2014) to date. It should also be noted that intracranial recordings have recently questioned whether transcranial electric stimulation can directly affect neuronal circuits, since traditional transcranial electric stimulation techniques require 4–6 mA to directly affect neuronal circuits (Vöröslakos et al., 2018), at least 16 times more than in the Voss et al. (2014) protocol. Therefore, it can be argued that the Voss et al. (2014) results were likely due to indirect mechanisms, i.e., the sensation of the electrotactile stimulus may have brought participants closer to waking up. This would increase cortical activation, particularly in key brain areas involved in LD (Mota-Rolim et al., 2008, 2010; Voss et al., 2009; Dresler et al., 2012; for review, see Baird et al., 2019), and therefore may have led to heightened dream consciousness.

## Conclusions and Perspectives

Most devices that were launched on crowdfunding platforms, mainly Aurora, iBand, and Neuroon, were able to raise much more resources than they asked for, which indicates that the public is interested in LD induction technologies. To date and to the authors' knowledge, the only research-ready equipment available in the market is ZMax; other devices, such as Neuroon, Aladdin, and LucidCatcher have had their release dates continually delayed. Only Neuroon and ZMax provide minimal technical information on how their algorithm detects REM sleep online, but none makes the data fully available. Most importantly, only DreamLight has been empirically tested with published results (Figure 1, table); thus, we conclude that better-controlled validation studies are necessary to prove the effectiveness of LD induction devices.

More scientific studies on other techniques to induce LD are also clearly warranted, and in particular, more reproducible studies in which LD can be induced. In a systematic review, Stumbrys et al. (2012) investigated 35 studies, which employed (a) cognitive techniques—such as autosuggestion, reality testing, and alpha feedback, for example (*n* = 26); (b) external stimulation—such as light, acoustic, and vibrotactile (*n* = 11); and (c) application of donepezil, which is an acetylcholinesterase inhibitor (*n* = 1). The authors observed that the methodological quality of the works analyzed was relatively low, and none of the induction techniques reported in these studies induced LD reliably and consistently. More research is needed to increase our understanding of external sensory stimulus processing during sleep and the conditions and the stimulus properties required for reliable dream content incubation, while preventing awakenings (Appel et al., 2018).

Promising results were obtained by two recent studies that applied galantamine (another acetylcholinesterase inhibitor), in combination with cognitive techniques, such as sleep interruption plus mnemonic induction of lucid dreams (MILDs; LaBerge et al., 2018a,b) or sleep interruption plus meditation and dream reliving (MDR; Sparrow et al., 2018). The association of the portable devices with cognitive and pharmacological techniques has great potential to improve the reliability of LD induction techniques.

## Author Contributions

All authors listed have made a substantial, direct and intellectual contribution to the work, and approved it for publication.

### Conflict of Interest Statement

AP worked for Inteliclinic, the company that developed Neuroon, for 3 months in 2017 as a consultant. The remaining authors declare that the research was conducted in the absence of any commercial or financial relationships that could be construed as a potential conflict of interest.

## Footnotes

^1^The TCP/IP socket is given according to the local network infrastructure policy for each installed device, and this could be static or dynamic. The required extended TCP/IP address information is not appropriated, as it depends on installation factors for each device.
